# The ability of an oral combination of afoxolaner, moxidectin and pyrantel to protect dogs from *Borrelia burgdorferi* infections transmitted by *Ixodes scapularis*

**DOI:** 10.1186/s13071-025-06753-8

**Published:** 2025-04-21

**Authors:** Joseph Prullage, Pascal Dumont, Arathy Nair, Manyun Liu, Utami DiCosty, Ricarda Süssenberger

**Affiliations:** 1 Boehringer Ingelheim Animal Health, Missouri Research Center, 6498 Jade Rd., Fulton, MO 65251 USA; 2 Boehringer Ingelheim Animal Health, Georgia Research Center, 3239 Satellite Boulevard, Duluth, GA 30096 USA; 3 TRS Labs, Inc., 215 Paradise Blvd, Athens, GA 30607 USA; 4 Boehringer Ingelheim Vetmedica GmbH, Binger Str. 173, 55216 Ingelheim am Rhein, Germany

**Keywords:** Canine, *Borrelia burgdorferi*, *Ixodes scapularis*, Prevention, Lyme borreliosis, Transmission

## Abstract

**Background:**

Two studies were conducted to determine whether treatment with NexGard^®^ Plus (NP), a combination of afoxolaner, moxidectin, and pyrantel, prevents transmission of *Borrelia burgdorferi* to dogs by naturally infected *Ixodes scapularis*.

**Methods:**

For each study, 20 dogs were randomly assigned to two groups (*n* = 10/group): NP and negative control. Twenty-eight days post-treatment, each dog was infested with approximately 50 *I. scapularis* that had a 60% *B. burgdorferi* infection rate in study 1 and a 38.5% infection rate in study 2. Five days post-infestation, ticks were counted and removed. The *B. burgdorferi*-specific C6 antibody was tested for using the SNAP^®^ 4Dx^®^ test (IDEXX) and the Lyme Quant C6 test with serum collected before treatment and infestation and 21, 35, 49, 63, and 75 days post-infestation. Skin biopsies were collected 76 days post-infestation and quantitative polymerase chain reaction (qPCR) conducted to detect *B. burgdorferi* DNA.

**Results:**

On the day of count and removal, no ticks were found on treated dogs, while control dogs had an average of 25.1 ticks in study 1 and 19.6 ticks in study 2, for efficacy of 100% (*P* ≤ 0.0001). All dogs were seronegative before infestation. The first dog in the control groups became seropositive 21 days post-infestation in study 1 and 35 days post-infestation in study 2 by the SNAP 4Dx test and by 21 days post-infestation by the Lyme Quant C6 test in both studies. Ten of 10 dogs in the control group in both studies seroconverted by the end of the study. None of the skin biopsies from treated dogs were positive for *B. burgdorferi* DNA, while at least three of the four skin biopsies from each of the control dogs tested positive at the end of the studies. No clinical signs of Lyme disease were detected in any of the dogs.

**Conclusions:**

The results of these studies indicate that NexGard^®^ Plus administered at a dose close to the minimum recommended dose of 2.5 mg/kg afoxolaner is effective 28 days after a single treatment in the prevention of *B. burgdorferi* transmission from naturally infected *I. scapularis* ticks to dogs.

**Graphical Abstract:**

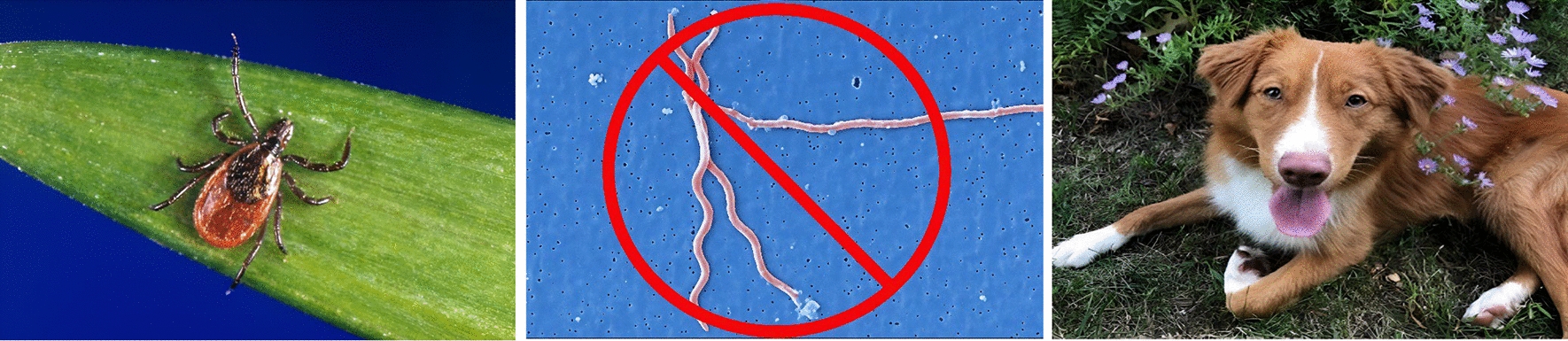

## Background

Lyme disease infection is a major threat to dogs throughout all states of the USA. Borreliosis is a well-recognized disease in humans but is not clearly defined in dogs [1]. Most infected dogs are subclinical, and it is difficult to correlate naturally acquired *Borrelia burgdorferi* infection with clinical signs in dogs such as fever, lameness, myalgia, and lethargy.

The efficacy of afoxolaner in controlling *Ixodes scapularis* infestations within 48 h for up to 1 month has been reported previously [2]. Baker et al. [3] demonstrated that afoxolaner alone (NexGard^®^, Boehringer Ingelheim) protected dogs from *B. burgdorferi* infection transmitted by wild *I. scapularis* ticks when using the same animal model and study design. The study design is aligned with current guidelines [4], has been successfully used with minor variations as to time points and number of ticks used for infestation, and can be considered a reliable model to demonstrate protective efficacy [5]. The transmission of *B. burgdorferi* from infected nymphs to experimental hosts has been extensively studied and reported as unlikely within the first 24 h after attachment, with the risk for transmission increasing over time [6–8]. Simultaneous feeding of infected ticks resulted in transmission within 48 h to approximately 50% of hosts, while transmission was less likely to be observed before 60 h for a single feeding tick [see [9] for a review]. Afoxolaner acts systemically and requires tick attachment and initial feeding before killing. Reducing the risk of pathogen transmission will therefore be achieved by killing ticks quickly before transmission occurs.

The aim of the paper and the studies presented herein is to contribute to the ongoing dialogue surrounding the mitigation of *Borrelia*-associated risks in the canine population.

## Methods

Dogs were acclimated to the facilities for at least 7 days and managed similarly and with due regard for their well-being. The study design was reviewed and approved by the sponsor’s and local institutional animal care and use committees and met United States Department of Agriculture Animal & Plant Health Inspection Service animal welfare requirements. The study was conducted in accordance with good clinical practices [10]. Standards included masking of personnel conducting efficacy evaluations and randomization to treatment groups. A physical examination was conducted during acclimation, and dogs were observed for general health at least once daily throughout the study.

**Fig. 1:**
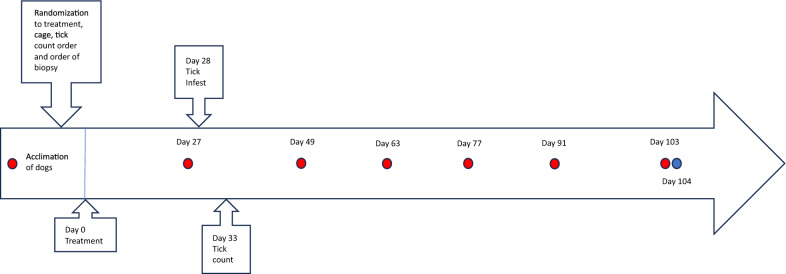
Timeline of the studies. Red dots = blood collections and blue dot = biopsy collections

Two studies were conducted that followed a single study design, as illustrated in Fig. 1. Twenty healthy purpose-bred Beagle dogs, 11 male and nine female per study, 7.4 to 8.2 months old and not previously exposed to ticks or treated with any ectoparasiticide drug were used in each study. They weighed 6.42 to 11.8 kg and were assigned to the two treatments completely at random. Ten dogs were assigned to the sham-dosed control group, and 10 dogs were assigned to the group treated with NexGard^®^ Plus (2.5 mg/kg afoxolaner, 12 µg/kg moxidectin, and 5 mg/kg pyrantel, Boehringer Ingelheim) in each study. A combination of chews was administered once in the fed state to provide a dose of afoxolaner, the acaricidal component, as close as possible to the minimum recommended dose of 2.5 mg/kg (study 1 = 2.80—3.72 mg/kg; study 2 = 2.53—3.31 mg/kg). Dogs were randomly assigned to cages, and random orders were used for tick counts and biopsy collections. The PLAN procedure in SAS version 9.4 was used for all randomizations. All personnel involved with evaluation of effectiveness (tick counts, serology, polymerase chain reaction [PCR] tests, and biopsy collection) and health observations were unaware of which treatment was administered.

Twenty-eight days post-treatment, each dog was infested with approximately 50 adult, unfed, wild-caught *I. scapularis* (approximately 25 males and 25 females) known to be infected with *B. burgdorferi* (Fig. 1). The ticks were left on the dogs for 5 days to maximize the chance of *B. burgdorferi* transmission. Thirty-three days post-treatment, study personnel blinded to treatment assignments examined all dogs for the presence of live ticks, which they counted and removed. The live or dead status of each tick was assessed after removal of the ticks. Once the entire body of the animal had been examined for ticks, the animal was combed using a flea comb to ensure that all ticks had been counted and removed.

On the day of tick removal, the four areas with the most ticks attached (i.e., congregations of two or more attached ticks, if available) were marked on a silhouette to identify the optimal sites for tissue biopsy collection. If ticks were not present, sites with evidence of prior tick attachment (if available) were marked. For animals with fewer than four attachment sites identified, biopsies were collected from the attachment sites identified most frequently on other dogs (neck area). All biopsies were collected from each dog on the same date in each study. The tick attachment sites were shaved and refreshed periodically during the study, as needed, to maintain identification of the selected sites.

Adult, unfed wild *I. scapularis* were collected in known endemic areas in Washington County, Rhode Island, for study 1, and Bluff Point State Park in Groton, Connecticut, and Branford, Connecticut, for study 2. PCR was used to determine the *B. burgdorferi* infection rate in the ticks based on a representative sample of 20 ticks in study 1 and 96 ticks in study 2 as previously published [11].

Blood was collected from each dog prior to treatment and 27 days after treatment to confirm that they were seronegative for *B. burgdorferi* prior to allocation, treatment, and infestation. Additional blood samples were collected as in Fig. 1. At each time point, the blood samples were placed in individually labeled serum separator tubes. Serum was recovered and tested using the IDEXX SNAP^®^4Dx^®^Test to detect C6 *B. burgdorferi* antibody, according to the manufacturer’s instructions. Serum samples were also tested using the Lyme Quant C6 test (IDEXX Bioresearch Laboratory). An animal was assessed as positive for the quantitative C6 test if its titer was ≥ 30 U/ml.

Skin biopsies were collected similarly to that of McCall et al. 2011 [12]. Each biopsy sample was placed in an individual 3 ml sterile cryovial container and chilled by being placed on ice immediately after collection. Biopsy samples were tested for presence of *B. burgdorferi* DNA by real-time quantitative PCR (qPCR) analysis. DNA from biopsy samples was extracted using the IndiMag Pathogen Kit (Indical). DNA purity and quantity were assessed by NanoDrop (Thermo Fisher Scientific). For the PCR reaction, the Indimix-JOE (Indical) master mix, along with previously designed primers and probe targeting *B. burgdorferi*, were utilized [13]. The PCR reaction was amplified using the 7500 Real-Time PCR platform (Applied Biosystems).

Efficacy with respect to live tick counts was calculated using the formula [(*C* − *T*)/*C*] × 100, where *C* is the arithmetic mean calculated from the least squares mean (LSMEAN) of the live tick counts for the control group, and *T* is the arithmetic mean calculated from the LSMEAN for the treated group.

The counts of the live ticks of the treated group were compared to the counts of the sham-dosed control group using an *F*-test. The MIXED procedure in SAS version 9.4 was used for the analysis, with treatment group listed as a fixed effect.

A dog was considered infected by *B. burgdorferi* if any of the following were true:

- a positive result on the SNAP 4Dx Plus test
- a positive result on the Lyme Quant C6 test
- a positive PCR test on one or more of the biopsy sites

The proportion of dogs infected with *B. burgdorferi* for the treated group was compared with the proportion of dogs infected in the control group by Fisher’s exact test, which was computed using the FREQ procedure in SAS version 9.4.

## Results

No live tick was found on any of the 10 dogs treated with NexGard^®^ Plus in either study. Live *I. scapularis* were recovered from all dogs in the control groups, with an average of 25.1 live ticks in study 1 (range 17–31) and 19.6 live ticks in study 2 (range 12–39). The single treatment provided 100% efficacy compared to the control group, and the population means of the two groups were significantly different (*P* ≤ 0.0001). The SNAP^®^4Dx^®^ and Lyme Quant C6 *B. burgdorferi* antibody test results are presented in Tables 1 and 2. All dogs in both studies were seronegative prior to allocation and treatment and prior to tick infestation. The first *Borrelia*-seropositive dog was observed in the control group 21 days post-infestation in study 1 and 35 days post-infestation in study 2 by the SNAP^®^4Dx^®^ test and by day 21 by the Lyme Quant C6 test in both studies. All 10 dogs in the control group in both studies seroconverted by the end of the study. None of the 10 NexGard^®^ Plus-treated dogs in either study became seropositive at any time during the study. In addition, none of the skin biopsies of the NexGard^®^ Plus-treated dogs were positive for *B. burgdorferi* by real-time qPCR, while at least three biopsies were positive from each of the control dogs in both studies. A dog was considered to be infected with *B. burgdorferi* if it had at least one positive result among the SNAP^®^ 4Dx^®^ Plus Test, Lyme Quant C6 Test, and *B. burgdorferi* PCR analysis. Using these criteria, 10 of 10 dogs in the control group and 0 of 10 of the NexGard^®^ Plus-treated dogs in both studies were infected. There was a significant difference in the expected proportion of infected dogs between the control and the treated groups (*P* < 0.0001).

No clinical signs of Lyme disease were detected in any of the dogs during the daily health observations conducted at least once daily through the end of the study.

**Table 1:** Serology results for *Borrelia burgdorferi* C6 antibodies in study 1

Study day^a^	SNAP 4Dx results		Lyme Quant C6 results	
	Control group 1^b^	NexGard Plus group 2^b^	Control group 1^b^	NexGard Plus group 2^b^
−7	0/10	0/10	0/10	0/10
27	0/10	0/10	0/10	0/10
49	4/10	0/10	7/10	0/10
63	3/10	0/10	10/10	0/10
77	9/10	0/10	10/10	0/10
91	10/10	0/10	10/10	0/10
103	10/10	0/10	10/10	0/10

^a^Study day 0 = day of treatment

^b^Number positive/number of dogs in the group

**Table 2:** Serology results for *Borrelia burgdorferi* C6 antibodies in study 2

Study day^a^	SNAP 4Dx results		Lyme Quant C6 results	
	Control group 1^b^	NexGard Plus group 2^b^	Control group 1^b^	NexGard Plus group 2^b^
−4	0/10	0/10	0/10	0/10
27	0/10	0/10	0/10	0/10
49	0/10	0/10	9/10	0/10
63	4/10	0/10	9/10	0/10
77	9/10	0/10	10/10	0/10
91	10/10	0/10	10/10	0/10
103	10/10	0/10	10/10	0/10

^a^Study day 0 = day of treatment

^b^Number positive/number of dogs in the group

## Discussion

No clinical signs of Lyme disease were seen in any of the dogs throughout the duration of the study. This was expected, as seropositive dogs naturally infected rarely demonstrate clinical manifestations that can unequivocally be linked to infection with *B. burgdorferi* [1].

The wild-caught ticks were viable as demonstrated by an excellent attachment rate in the sham-dosed control animals of 50% overall (range 34–62%) in study 1 and 39% overall (range 24–78%) in study 2. The requirements for tick retention as described in current guidelines were met [14]. Together with the tick infectivity of 60% in study 1 and 39% in study 2, an appropriate challenge was obtained. This is supported by the demonstration of *B. burgdorferi* infection in 10 of 10 dogs in the sham-dosed control group in both studies by detecting dogs that acquired the C6 peptide of *B. burgdorferi*. The IDEXX SNAP^®^ 4Dx^®^ test employed for serology is used extensively in the field, and its performance with regard to detection of C6 *B. burgdorferi* antibodies has been demonstrated, i.e., sensitivity of 96.7% and specificity of 98.8% [15]. The IDEXX Lyme Quant C6 test was used as an additional test for seroconversion, as it will generally detect antibodies to the C6 antigen earlier than the qualitative SNAP^®^ 4Dx^®^ test [16]. In addition, the skin biopsies were positive for *B. burgdorferi* by real-time qPCR in at least three biopsies from each of the control dogs in both studies.

## Conclusion

The results of this study indicate that NexGard^®^ Plus administered at a dose close to the minimum recommended dose of 2.5 mg/kg afoxolaner is effective 28 days after a single treatment in the prevention of Lyme borreliosis transmission from naturally infected *I. scapularis* ticks to dogs.

## Data Availability

All data supporting the research reported in this article are reported within the article.

## Abbreviation

LSMEAN: Least squares mean
